# Exercise-induced cardiac remodeling in non-elite endurance athletes: Comparison of 2-tiered and 4-tiered classification of left ventricular hypertrophy

**DOI:** 10.1371/journal.pone.0193203

**Published:** 2018-02-20

**Authors:** Lukas D. Trachsel, Christoph P. Ryffel, Stefano De Marchi, Christian Seiler, Nicolas Brugger, Prisca Eser, Matthias Wilhelm

**Affiliations:** Preventive Cardiology & Sports Medicine, University Clinic for Cardiology, Inselspital, Bern University Hospital, University of Bern, Bern, Switzerland; Michigan State University, UNITED STATES

## Abstract

**Background:**

Long-term endurance sport practice leads to eccentric left ventricular hypertrophy (LVH). We aimed to compare the new 4-tiered classification (4TC) for LVH with the established 2-tiered classification (2TC) in a cohort of normotensive non-elite endurance athletes.

**Methods:**

Male participants of a 10-mile race were recruited and included when blood pressure (BP) was normal (<140/90 mmHg). Phenotypic characterization of LVH was based on relative wall thickness (2TC), and on LV concentricity^2/3^ (LV mass/end-diastolic volume [LVM/EDV]^2/3^) plus LVEDV index (4TC). Parameters of LV geometry, BP, cumulative training hours, and race time were compared between 2TC and 4TC by analysis of variance, and post-hoc analysis.

**Results:**

Of 198 athletes recruited, 174 were included. Mean age was 41.6±7.5 years. Forty-two (24%) athletes had LVH. Allocation in the 2TC was: 32 (76%) eccentric LVH and 10 (24%) concentric LVH. Using the 4TC 12 were reclassified to concentric LVH, and 2 to eccentric LVH, resulting in 22 (52%) eccentric LVH (7 non-dilated, 15 dilated), and 20 (48%) concentric LVH (all non-dilated). Based on the 2TC, markers of endurance training did not differ between eccentric and concentric LVH. Based on the 4TC, athletes with eccentric LVH had more cumulative training hours and faster race times, with highest values thereof in athletes with eccentric dilated LVH.

**Conclusions:**

In our cohort of normotensive endurance athletes, the new 4TC demonstrated a superior discrimination of exercise-induced LVH patterns, compared to the established 2TC, most likely because it takes three-dimensional information of the ventricular geometry into account.

## Introduction

Left ventricular hypertrophy (LVH) is associated with cardiovascular morbidity and mortality in hypertensive patients as well as in the general population [1–3]. Dilatation of the LV is associated with poor prognosis in hypertensive patients [4]. However, LVH reflects a physiological adaptation to exercise in athletic individuals [5], where it conveys an excellent prognosis [6]. LV mass can increase through LV cavity dilatation, wall thickening, or combinations thereof. Exercise-induced LV remodeling appears to be a phasic phenomenon, where the increase in LV cavity size may precede or follow the increase in LV wall thickness, based on type and level of sports, finally leading to eccentric LVH [7, 8]. Traditionally, M-mode echocardiography has been used to quantify LV mass. LVH is sub-classified based on a high or low relative wall thickness (RWT), leading to a 2-tiered classification (2TC) of LVH (concentric or eccentric)[9]. Recently, a more distinct classification of LV geometry has been developed, taking three-dimensional information of the LV into account. A 4-tiered classification (4TC) of LVH was developed for MRI based on the presence or absence of increased LV concentricity^2/3^ (LV mass/end-diastolic volume (EDV)^2/3^) and the presence or absence of increased LV EDV index [10]. Compared to the 2TC, the 4TC demonstrated superior risk-stratification for adverse cardiovascular events in hypertensive patients (with the method adapted to echocardiography [4]) and also in the general population [4, 11]. A recent study in 1,083 healthy elite white athletes (41% female; mean age 21.8±5.7 years) found that based on the 2TC, 4% of female and 15% of male athletes competing in dynamic sports demonstrated LV concentric remodeling or hypertrophy. The authors concluded that these patterns may be seen as marker of disease [12]. The new 4TC may contribute to a better risk stratification of athletes with LVH. Therefore, the aim of the present study was to compare the new 4TC of LVH with the established 2TC in a cohort of normotensive non-elite endurance athletes with a wide range of training volume and competitive race performance. Classifications were compared with regard to their power to discriminate markers of endurance training, namely cumulative lifetime training hours and race time.

## Methods

### Participants and protocol

The Grand Prix of Bern is a popular 10 mile race in Switzerland with over 30,000 participants annually. Male Caucasian non-elite runners were recruited during the 2010^th^ and 2011^th^ events for two studies on endurance exercise and atrial remodeling as previously described [13, 14]. In addition, athletes with a history of high volume endurance training for many years were selected from the 2015^th^ event. We included runners aged 30 years and older, with and without a history of former long-distance runs. We excluded athletes with a history of cardiovascular disease, regular medication intake (incl. non-steroidal anti-inflammatory drugs), or cardiovascular risk factors, in particular arterial hypertension, defined as an office blood pressure (BP) ≥140/90 mmHg at the initial visit [15].

Body weight was measured with light clothing using a digital-balanced scale and stature using a wall-mounted stadiometer. Body mass index (BMI) was calculated as body mass divided by stature squared (kg^.^m^-2^). Body surface area (BSA) was calculated using the DuBois formula [16]. Resting heart rate and BP were measured in a quiet room after 5 min in supine position shortly before the transthoracic echocardiography (TTE). BP measurements were performed according to the guidelines: BP was measured once in both arms to detect possible differences with an oscillometric device (Dinamap XL; Criticon Inc., Tampa, Florida, USA). We chose the arm with the higher value as the measuring arm. Then, BP was measured two additional times at the chosen arm and the mean of these measurements used for data analysis [15].

Further, athletes completed a comprehensive questionnaire to ascertain the personal and sports history. The calculation of average training hours was determined by the athletes’ estimation and/or exercise diary. The variable “training years” included only training years from age 18 years onward. Lifetime training hours were calculated using the following formula: average endurance training hours per week x 52 x training years [13, 14]. Official 10 mile race time was taken from the Grand Prix of Berne database of the study participation year. Cumulative lifetime training hours and 10 mile race time served as markers for the quantity and quality of endurance training. All athletes provided written informed consent and the protocol was approved by the ethics committee of the Canton of Berne.

### Transthoracic echocardiography

Standard two-dimensional TTE was performed on a Phillips iE33 System (X5-1 transducer, Phillips Medical Systems, Zurich, Switzerland). LV internal diameter (LVID), interventricular septum (IVS) and posterior wall thickness (PWT) were measured in M-mode from the parasternal long-axis view at end-diastole. LV mass was calculated based on the cube formula: LV mass = 0.8 x 1.04 x [(IVS + LVID + PWT)^3^ –LVID^3^] + 0.6 g and indexed for BSA [9].

LV end-diastolic (LVEDV), and LV end-systolic volumes (LVESV) were calculated using the biplane method of disks summation technique. Volume measurements were based on tracings of the blood-tissue interface in the apical four- and two-chamber views. At the mitral valve level, the contour was closed by connecting the two opposite sections of the mitral ring with a straight line. LV length was measured from this line to the apical point of the LV contour. All volumes were indexed for BSA [9].

**Fig 1:**
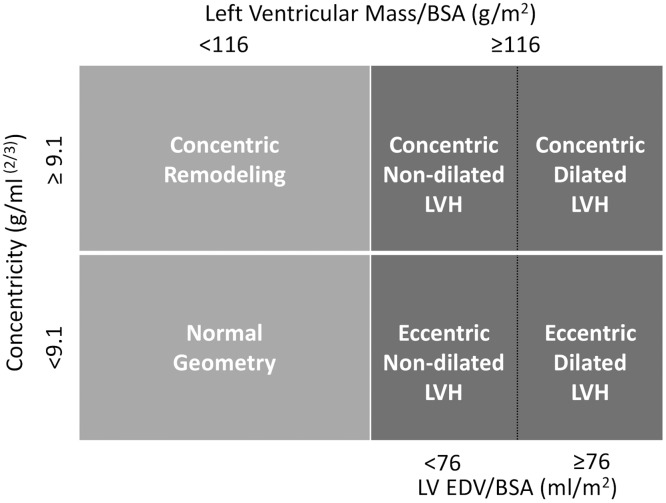
Schematic illustration of the four-tiered classification of left ventricular hypertrophy. LV EDV indicates left ventricular end-diastolic volume; BSA, body surface area; and LVH, left ventricular hypertrophy.

LVH was defined as LV mass/BSA≥116 g/m^2^ for both, the 2TC and 4TC systems [4, 9]. For the 2TC, relative wall thickness (RWT) was calculated as 2 x PWT/LVID. The threshold for concentric remodeling/concentric hypertrophy was set at RWT>0.42 [9]. For the 4TC, left ventricular geometry was specified by LV concentricity (LV mass/LVEDV^2/3^) and LVEDV/BSA as recently proposed by Khouri and co-workers [10]. Echocardiographic thresholds for increased concentricity were ≥9.1g/ml^2/3^ and for increased LVEDV/BSA ≥76 ml/m^2^ (Fig 1) [4].

LV systolic function was expressed as ejection fraction (EF), derived from the LVEDV and LVESV [9]. LV diastolic function was assessed by pulse-wave and tissue Doppler in the apical four-chamber view. Peak early filling (E wave) and late diastolic filling (A wave) velocities, the E/A ratio, peak early and late diastolic velocities at the septal and lateral side of the mitral annulus were recorded and the mean value was calculated and defined as *E’* mean and *A’* mean. Diastolic dysfunction was defined as *E’* septal <8 cm/s and/or *E’* lateral <10 cm/s [9].

### Data analysis

The data was analysed with SPSS Software for Windows (Version 17.0, SPSS Inc., Chicago, USA). The normality of quantitative variables was inspected visually and homogeneity of variances tested by Levene’s test. Normally distributed data were presented as mean ± standard deviation (SD), and non-normally distributed variables as median (Interquartile range).

Two linear regression models were performed with LV mass index as dependent variable and cumulative lifetime training hours or 10 mile race time as independent variables, because of their strong inter-correlation. In addition, systolic BP, age and BMI were used as independent variables in both models, with forced inclusion. Alpha was set at 0.05.

All athletes were classified by the 2TC and 4TC system. Subsequently, athletes were grouped with regard to the 2TC and 4TC allocation, and variables reflecting the main geometric LV properties were compared between resulting groups separately for groups with or without LVH. For between group differences the following variables were tested: age, BMI, BSA, resting HR, resting systolic and diastolic BP, cumulative lifetime training hours, 10 mile race time, and all measured echocardiographic variables. Normally distributed variables with homogenous variances were compared between groups of the 2TC and 4TC system by univariate ANOVA with Tukey post-hoc testing or Dunnet post-hoc testing if variances were heterogeneous. Data with non-parametric distribution were compared between groups by Kruskal-Wallis tests with Bonferroni-adjusted Mann-Whitney-U post-hoc testing.

## Results

### Participant characteristics

A total of 198 male runners were recruited (2010: n = 70, 2011: n = 108, 2015: n = 20). Twenty four runners had to be excluded (14 had undiagnosed arterial hypertension, 8 could not participate in the race because of muscular problems, 1 had mitral valve prolapse, and 1 had hypercholesterolemia). Although we had 17 normotensive athletes with an interventricular septum diameter between 12 mm and 15 mm, none of them had additional clinical features of hypertrophic cardiomyopathy[17].

Thus, data of 174 normotensive Caucasian male runners was included in the analyses. Mean age was 41.6±7.5 years. A broad spectrum of endurance athletes was covered, ranging from leisure-time runners with a first participation in a 10 mile race, up to semi-professional long-distance runners with an average of 15 weekly endurance training hours (median 4, interquartile range 4), and more than 17,000 cumulative lifetime training hours (median 3246, interquartile range 5857).

### Statistical models

**Fig 2:**
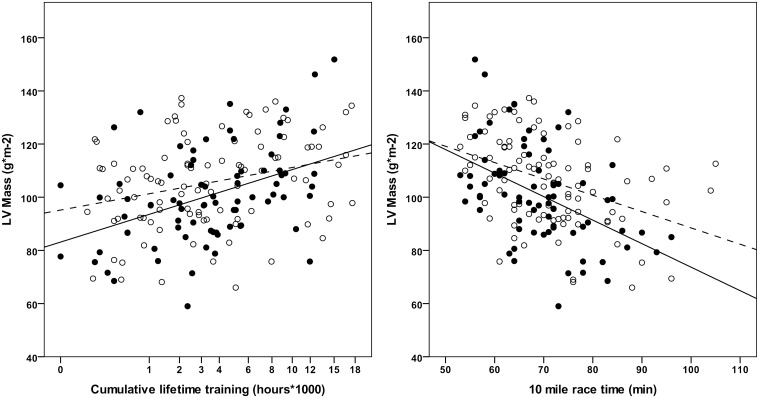
Impact of cumulative lifetime endurance training and 10 mile race time on LV mass illustrated for normal (<120/80 mmHg, filled circles) and non-optimal (120/80-139/89 mmHg, empty circles) blood pressure. Solid line indicates linear regression for athletes with normal blood pressure and dashed line linear regression for athletes with non-optimal blood pressure.

There was a significant inverse correlation between cumulative lifetime training hours and 10 mile race time (r = -0.566; P<0.001), and both cumulative lifetime training hours and 10 mile race time were associated with LV mass index (Fig 2). The linear regression model for LV mass index, including cumulative lifetime training hours together with age, BMI, and systolic BP, explained 14% of the total variance. Standardised beta coefficients were 0.316 (p<0.001) for cumulative lifetime training hours, and 0.169 (p = 0.023) for systolic BP. The linear regression model for LV mass index, including 10 mile race time together with age, BMI, and systolic BP, explained 21% of the total variance. Standardised beta coefficients were -0.469 (p<0.001) for 10 mile race time and 0.188 (p = 0.009) for systolic BP. Age and BMI had no impact on LV mass index in both models. Fig 2 shows the correlation of cumulative lifetime training hours, and 10 mile race time with LV mass index, stratified for optimal (<120/80 mmHg) and non-optimal (120-139/80-89 mmHg) BP.

### Characteristics of classes according to 2-tired and 4-tired classification

Characteristics of endurance athletes, stratified according to the conventional 2TC and the new 4TC are shown in Tables 1 and 2. Forty-two athletes (24%) had LVH. In the 4TC only five groups are presented, as no athlete was classified as concentric dilated LVH. In both classification systems significant differences between groups were found for resting HR, cumulative lifetime training hours, 10 mile race time, and most echocardiographic LV geometric parameters. Age, BSA and BP differed significantly between groups only in the 4TC.

**Table 1:** Characteristics of endurance athletes, stratified according to the conventional 2-tiered classification of LVH.

Variable	Normal LV geometry (n = 98)	Concentric LV remodeling (n = 34)	Eccentric LVH (n = 32)	Concentric LVH (n = 10)	P-Value
Baseline characteristics					
Age (years)	41±7	42±6	41±7	47±11	0.100*
Body mass index (kg/m^2^)	23.0±1.2	23.3±2.0	22.7±1.3	22.6±2.4	0.581
Body surface area (m^2^)	1.9±0.1	1.9±0.1	1.9±0.1	1.9±0.1	0.203
Heart rate at rest (bpm)	60±10	69±11†	55±10	61±6	<0.001
Systolic BP at rest (mmHg)	122±8	121±9	124±10	124±11	0.668
Diastolic BP at rest (mmHg)	76±6	76±8	77±7	80±4	0.441
Markers of endurance training					
Cumulative lifetime endurance training (hours)	2600 (1040, 5460)	2838 (953, 5005)	6552 (2262, 10616)	5720 (2080, 9152)	0.005
10 mile race time (hours:min)	1:12±0:11	1:13±0:10	1:04±0:07	1:06±0:09	0.001
Echocardiography					
LV mass/BSA (g/m^2^)	95.1±13.2	98.1±10.7	127.7±8.2	125.4±6.0	b.d.
Relative wall thickness	0.35±0.04	0.48±0.05	0.37±0.04	0.46±0.02	b.d.
LV EDV/BSA (ml/m^2^)	60.1±10.7	52.5±8.5†	72.3±10.9	56.4±13.1‡	<0.001
LV length (cm)	8.8±0.7	8.8±0.6	9.1±0.6	8.5±0.7‡	0.42
IVS (mm)	10.1±1.2	11.2±1.0†	11.4±1.1	12.3±1.3‡	<0.001
LVID (mm)	51.7±3.5	46.5±3.4†	55.7±2.9	50.7±2.6‡	<0.001
PWT (mm)	9.1±0.9	11.1±0.7†	10.1±0.8	11.6±0.5‡	<0.001
LV ejection fraction (%)	64.0±4.9	64.0±5.2	64.7±5.2	65.1±5.5	0.779
E’ mean (cm/s)	12.6±2.0	13.0±1.5	12.5±1.8	11.5±2.3	0.190
LAVI (ml/m^2^)	30.3±5.7	28.2±6.6	35.0±6.6	33.9±7.1	<0.001

*P-Value of Dunnett-Test because of heterogeneity of variances

^†^P-Value of Tukey post-hoc test <0.05 vs normal LV mass

^‡^P-Value of Tukey post-hoc test <0.05 vs eccentric LVH

BP, blood pressure; LV, left ventricle; BSA, body surface area; IVS, interventricular septum; ID, internal diameter; PWT, posterior wall thickness; EDV, end-diastolic volume; E’ mean, *E‘*, peak early diastolic septal and lateral tissue Doppler mitral annular velocity; LAVI: left atrial volume index.

**Table 2:** Characteristics of endurance athletes, stratified according to the new 4-tiered classification of LVH.

Variable	Normal LV geometry (n = 104)	Concentric LV remodeling (n = 28)	Eccentric LVH non-dilated (n = 7)	Eccentric LVH dilated (n = 15)	Concentric LVH non-dilated (n = 20)	P-Value
Baseline characteristics						
Age (years)	41±7	44±7†	39±5	39±7	46±10‡,§	0.019
Body mass index (kg/m^2^)	22.9±1.9	23.8±1.9†	22.6±1.5	22.4±1.3	22.9±1.8	0.133
Body surface area (m^2^)	1.9±0.1	2.0±0.1	1.8±0.1	1.9±0.1	1.9±0.2	0.024
Heart rate at rest (bpm)	61±10	68±10†	52±6	51±8	62±9§	<0.001
Systolic BP at rest (mmHg)	121±9	127±7†	125±11	124±10	124±10	0.040
Diastolic BP at rest (mmHg)	76±7	78±7	78±5	74±8	80±6§	0.040
Markers of endurance training						
Cumulative lifetime endurance training (hours)	2600 (1040, 5304)	2838 (884, 5200)	7020 (4966, 8554)	9532 (8086, 13282)	2340§ (1690, 5720)	<0.001
10 mile race time (hours:min)	1:11±0:10	1:15±0:13	1:02±0:04	1:00±0:05	1:08±0:08	<0.001*
Echocardiography						
LV mass/BSA (g/m^2^)	93.3±12.7	105.1±7.2†	120.0±2.2	129.3±8.0	128.0±7.6‡	b.d.
Concentricity (ml/m^2/3^)	7.5±0.9	10.0±0.9	8.6±0.3	8.5±0.5	10.7±1.3	b.d.
LV EDV/BSA (ml/m^2^)	60.9±10.0	48.2±6.2†	70.5±3.0	81.8±7.1	57.9±8.8‡,§	b.d.
LV length (cm)	8.8±0.7	8.6±0.6	9.2±0.6	9.4±0.5	8.5±0.5‡,§	<0.001
IVSd (mm)	10.2±1.2	11.4±1.0†	10.5±0.6	11.2±1.2	12.1±1.2‡,§	<0.001
LVID (mm)	50.4±4.1	50.2±4.3	53.0±2.0	57.3±2.5	52.9±4.0§	<0.001
PWT (mm)	9.4±1.1	10.5±1.0†	10.4±1.0	10.0±1.0	10.9±0.7§	<0.001
LV ejection fraction (%)	63.7±4.7	64.9±4.8	64.5±5.2	63.7±4.6	65.9±5.7	0.368
E’ mean (cm/s)	12.8±2.0	12.5±1.7	12.6±1.3	12.3±2.0	12.1±2.1	0.640
LAVI (ml/m^2^)	30.1±6.0	28.5±5.9	34.4±4.3	36.3±7.1	33.8±7.0	<0.001

*P-Value of Dunnett-Test because of heterogeneity of variances

^†^P-Value of Tukey post-hoc test <0.05 vs normal LV mass

^‡^P-Value of Tukey post-hoc test <0.05 vs eccentric non-dilated

^§^P-Value of Tukey post-hoc test <0.05 vs eccentric dilated

BP, blood pressure; LV, left ventricle; BSA, body surface area; IVS, interventricular septum; ID, internal diameter; PWT, posterior wall thickness; EDV, end-diastolic volume; E’ mean, *E‘*, peak early diastolic septal and lateral tissue Doppler mitral annular velocity; LAVI: left atrial volume index.

In both the 2TC and the 4TC, athletes with LVH had significant more training hours and a faster 10 mile race time compared to athletes with normal LV geometry or concentric LV remodeling. Based on the 2TC, markers of endurance training did not differ between eccentric and concentric LVH. Based on the 4TC, athletes with eccentric LVH had more cumulative training hours, a faster race time and lower HR. Highest values were observed in athletes with eccentric dilated LVH.

### Discrepancies between 2-tired and 4-tired classification

**Fig 3:**
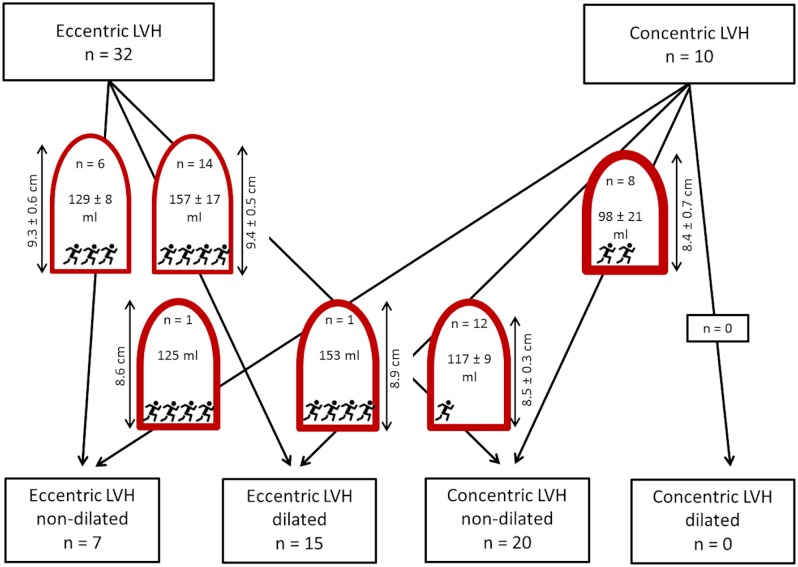
Number of athletes in each tier of the 2TC and 4TC, as well as number of athletes who were reclassified from 2TC to 4TC. LV end-diastolic volume and LV length are indicated inside or next to the schematic representations of the ventricles (mean ± SD). Training characteristics are indicated by the number of runner symbols (cumulative lifetime training (hours)/10 mile race time (min): 1 runner: <4000 and/or ≥70; 2 runners: 4000 to 5999 and/or 65 to 69; 3 runners: 6000–7999 and/or 60 to 64; 4 runners: ≥8000 and/or <60).

In athletes without LVH, the majority of those with normal LV geometry according to the 2TC remained in the group with normal LV geometry according to the 4TC system (83 of 98, 85%), while 15 athletes (15%) were reclassified to the group of concentric remodeling. These 15 athletes had significantly greater PWT, smaller LVEDV, and a higher resting systolic BP. Of the athletes in the concentric remodeling group according to the 2TC, 21 of 34 (62%) were reclassified to normal LV geometry in the 4TC. Compared to those remaining in the concentric remodeling group, they had significantly greater LV length, greater LVEDV and lower systolic BP (Fig 3, top row).

From the 32 athletes with eccentric LVH according to the 2TC, 6 (19%) were classified to eccentric non-dilated LVH, and 14 (44%) to eccentric dilated LVH in the 4TC. Twelve athletes (38%) were reclassified to concentric non-dilated LVH. The 12 athletes who were no longer classified as eccentric LVH in the 4TC system differed significantly from those with eccentric LVH in that they had shorter LV length, smaller LVEDV, greater PWT, less cumulative endurance training hours, and a slower 10 mile race time. The majority of athletes classified into the concentric LVH group according to the 2TC system remained in the 4TC concentric non-dilated LVH group (8 of 10, 80%). One athlete was reclassified to eccentric non-dilated LVH, and one to eccentric dilated LVH. These athletes differed from those with concentric non-dilated LVH in that they had a longer LV length, larger LVEDV, smaller PWT, more cumulative lifetime training hours, and a faster 10 mile race time (Fig 3, bottom row).

## Discussion

In our cohort of non-elite endurance athletes we compared the established 2TC of LVH with the recently proposed 4TC. This classification has been introduced using magnetic resonance imaging data of the LV [10]. Usefulness and superior risk-stratification over the conventional 2TC has been demonstrated in the general population [11]. Comparable results have been shown in patients with arterial hypertension using TTE [4].

To the best of our knowledge this is the first study demonstrating to better distinguish between exercise-induced LVH patterns and pathological LVH with the new 4TC. In our cohort of normotensive non-elite Caucasian endurance runners, one in four athletes presented with LVH. As both classifications use the same LV mass index threshold for LVH, both classifications discriminated equally well between low and high markers of endurance training, namely cumulative lifetime training hours and 10 mile race time. In the group of athletes with LVH, only the 4TC discriminated these markers further between different patterns of LVH. Athletes with eccentric LVH had more cumulative training hours and a faster 10 mile race time, compared to athletes with concentric LVH. Compared to athletes with eccentric non-dilated LVH, markers of endurance training were more pronounced in athletes with eccentric dilated LVH.

**Fig 4:**
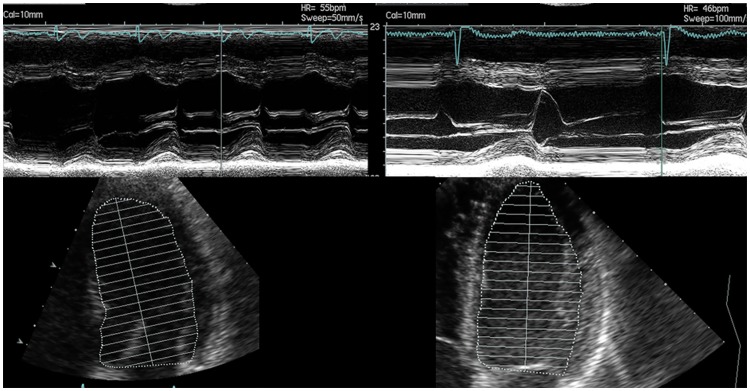
Echocardiography images of M-mode and apical two-chamber views. Athlete 1 was classified as eccentric LVH with 2TC due to a LVID of 57.1 mm and a PWT of 9.8 mm resulting in a relative wall thickness of 0.34. He was reclassified with 4TC to concentric non-dilated LVH due to a relative short LV length of 8.2 cm and an associated LV EDV/BSA of 60.1 ml/m^2^ resulting in a Concentricity of 10.4 ml/m^2/3^. Athlete 2 was also classified as eccentric LVH with 2TC due to a LVID of 53.6 mm and a PWT of 10.6 mm resulting in a relative wall thickness of 0.40. In contrast to Athlete 1, Athlete 2 was reclassified with 4TC to eccentric non-dilated LVH due to a longer LV length of 9.3 cm and an associated higher LV EDV/BSA of 71.5 ml/m^2^ resulting in a smaller Concentricity of 8.7 ml/m^2/3^.

In the 2TC the characterization of LVH patterns is based on the RWT from M-mode measurements, using one dimension only [9]. Therefore the 2TC cannot discriminate between longer and shorter ventricles. In contrast, the 4TC uses LV concentricity, describing the ratio of LV mass/LVEDV^2/3^ [10]. The LVEDV includes three-dimensional information of the LV cavity, including LV length. In a longitudinal study in young, competitive athletes (male rowers, mean age 18.6±0.5 years), acute exercise exposure (3 months) led to an increase of LV mass by 14%, mainly driven by an increase in LVEDV (+11%), followed by an increase in wall thickness (+2%), resulting in an eccentric pattern of LV remodeling. Chronic exercise exposure (36 months) led to a further increase in LV mass by 9%, now mainly driven by an increase in LV wall thickness (+11%), followed by an increase in LV length (+5.8%), associated with a further increase in LVEDV (+2%), and maintaining an eccentric pattern of LVH[7]. In agreement with this observation a MRI study in marathon runners and patients with mitral regurgitation has been pointed out, that only endurance athletes show a proportional enlargement of all three cavity dimensions while maintaining a normal LV mass/volume ratio. In contrast, pathological enlargement, such as found in chronic volume overload e.g. mitral regurgitation, has been shown to lead to an enlargement of the short axis of the LV only, without concomitant enlargement of the long axis length[18]. This illustrates the importance of setting LV mass and wall thickness in relation to LV cavity volume rather than the short axis diameter only. Especially for the correct discrimination between the physiological adaptation of the athlete’s heart and pathological wall thickening that is over-proportional to cavity volume, e.g. in the presence of an elevated BP. The 4TC justly reclassified 12 athletes with low training volume and/or slow race time from eccentric LVH (2TC) to concentric non-dilated LVH due to their thickened walls concomitant with smaller LV cavity volume and length (Fig 4). This suggests that the 4TC may be more sensitive with regard to identifying concentric non-dilated LVH. On the other hand, two athletes with concentric LVH based on the 2TC but high markers of endurance training were justly reclassified as non-dilated (1) and dilated (1) eccentric LVH due to their larger LV cavity volume and length. The 4TC eccentric dilated and non-dilated LVH groups reflect the typical athlete’s heart with large LVEDV as a consequence of a large number of cumulative training hours[19].

### Limitations

Our cohort comprised of athletes in whom normotension was defined based on one office BP measurement only. BP as a continuous variable remained a weak but independent predictor of LV mass index. 24-h BP measurements may have improved the selection of normotensive athletes. We demonstrated recently in another study that 38% of middle-aged endurance athletes with a normal office BP fulfilled criteria for masked hypertension based on 24-h ambulatory BP measurements. The relative risk of masked hypertension was increased two-fold in the presence of a non-optimal office BP(≥120/80mmHg), and masked hypertension was associated with a higher LV mass/volume ratio [20]. Thus, some of our athletes may have had masked hypertension with a likely impact on LV geometry.

Further, cumulative endurance training hours were based on estimation and prone to recall bias. However, we demonstrated a good inverse correlation with the precisely measured 10-mile race time, and both markers of endurance training demonstrated similar results. Finally, two-dimensional echocardiography has known limitations in assessing LV mass and volume[9], although the potential of the 4TC for risk stratification using cut-offs derived from two-dimensional echocardiography has been shown in patients with arterial hypertension [4]. Finally, medication data was based on patients’ answers only.

### Conclusions

In our cohort of normotensive endurance athletes, the new 4TC demonstrated a superior discrimination of exercise-induced LVH patterns, compared to the established 2TC, most likely because it takes three-dimensional information of the LV geometry into account.

## Supporting information

S1 Data Raw data of all analysed data. (SAV)
